# Emotional Changes and Functional Progressions during Postoperative Rehabilitation in Collegiate Student-Athletes: A Preliminary Study

**DOI:** 10.3390/healthcare9020184

**Published:** 2021-02-09

**Authors:** Seo Young Lee, Jihong Park

**Affiliations:** 1 Athletic Training Laboratory, Department of Physical Education, Graduate School of Education, Kyung Hee University, Yongin 17104, Korea; lsyoung85@khu.ac.kr; 2 Athletic Training Laboratory, Department of Sports Medicine, Kyung Hee University, Yongin 17104, Korea

**Keywords:** athletic injury, emotion, visual analogue scale, Lower-extremity Functional Scale

## Abstract

An interrelationship between psychological and physical health is generally accepted in the field of sports medicine. This preliminary study explored the association between emotional changes and functional outcomes and aimed to describe how each aspect progresses during postoperative rehabilitation. Four collegiate student-athletes (1 female and 3 males) who underwent supervised postoperative rehabilitation due to a lower-extremity injury volunteered for participation in the study. Emotion was quantified using a visual analogue scale prior to and after each session while self-reported function using the Lower-extremity Functional Scale was assessed every eight sessions throughout rehabilitation. There was a moderate correlation between emotional changes and functional outcomes (*r* = 0.58, *p* < 0.0001). After the first emotional improvement, patients experienced six emotional deteriorations (28% of the entire rehabilitation period; F_49,297_ = 2.25, *p* < 0.0001), while their function consistently increased (F_49,147_ = 17.39, *p* < 0.0001). Clinicians should be aware of the relationship between emotional changes and functional progression as well as the occurrence of emotional fluctuations when supervising and consulting patients during postoperative rehabilitation. A larger study is warranted to generalize the results.

## 1. Introduction

Sports injuries often require surgical intervention. During postoperative rehabilitation, patients (athletes) experience negative psychological responses such as frustration and self-doubt [1,2]. The quantity and quality of these psychological challenges are determined by several contributing factors such as cognitive appraisal and behavioral and emotional responses, together with both internal (personal) and external (situational) factors [3]. Functional progressions (e.g., pain reduction or mobility gain) and/or events occurring outside the rehabilitation space (e.g., socializing with family and friends) can have positive effects on the patient’s psychological responses [4]. Among the interactions involving each factor listed by Wiese [3], changes in emotional responses appear to impact behaviors such as adherence which in turn have been found to impact on rehabilitation outcomes [5]. Emotion is an inconsistent and unpredictable, temporary psychological state [6]; thus, emotional fluctuations (e.g., change from negative to positive or vice versa) are common psychological responses that may occur throughout the rehabilitation period.

The interactive relationship between emotional status and functional outcomes is generally accepted in the field of sports medicine. For example, individuals with higher degrees of psychological well-being display a better immune function [7] or physical health [8]. This pattern has also been reported in postoperative rehabilitation in that successful rehabilitation outcomes are linearly correlated with positive emotions in patients following anterior cruciate ligament reconstruction [9]. If emotional status is one of the contributing factors to functional restoration and progression, knowledge of patients’ emotional changes within a single session as well as throughout the rehabilitation period would be beneficial for clinicians as they supervise and consult these individuals. As emotion is typically multidimensional [6] and may only last for as few seconds to minutes [10], identifying change in the emotional response throughout the rehabilitation period is very difficult. While the best way to assess emotion has not been determined, self-report measure is one of the valid and recommended measurement tool [11]. Assessing the emotional status before and after rehabilitation using a simple quantification tool such as visual analogue scale (VAS) would help us obtain patients’ emotional fluctuation over small periods of time. 

The major components of postoperative rehabilitation generally consist of aerobic, flexibility, resistance, and balance exercises in conjunction with therapeutic modalities [12]. The rehabilitative interventions of these components are progressively administered throughout the stages of the acute, recovery, and functional improvement [13]. Although the necessity of psychological interventions to enhance the process of rehabilitation is widely accepted, clinicians often feel less confident in understanding patients’ emotions and implementing psychological interventions [14]. One of the previously proposed conceptual models during a rehabilitation process after sports injuries was the ‘normal rehabilitation curve’ [15]. According to this concept, patients experience a cycle of emotional ups (positive) and downs (negative) during the ongoing process of functional restoration in rehabilitation. Important psychological factors in postoperative rehabilitation such as motivation, commitment and adherence can be affected by daily emotional fluctuations, also possibly influencing functional outcomes [16,17]. This concept is clinically meaningful because education on this curve would help patients to stay motivated and progressing [15]. If the emotional curve during rehabilitation progresses has several downslopes, consulting strategies inducing and guiding these changes would be necessary. However, the existence of emotional fluctuations has not been objectively examined.

Therefore, in this preliminary study, we sought to describe the association between emotional changes and functional outcomes and to describe how each aspect progresses during the course of postoperative rehabilitation. Emotion was quantified using a 100 mm VAS [18] before and after each rehabilitation session. Function was assessed using the Lower-extremity Functional Scale (LEFS) [19] every eight sessions throughout rehabilitation period. Specifically, we asked: (1) how is emotion (obtained before or after each rehabilitation session or overall: time-collapsed) related to level of function?; (2) can a single session of rehabilitation affect emotion?; and (3) after the first occurrence of emotional or functional improvement, would patients experience emotional or functional decrement during rehabilitation? We hypothesized a moderate to strong relationship between emotion and function and that a single rehabilitation session would be associated with in emotional improvement. After the first upswing, emotion would involve several downs though the same would not be true for function, which continue to increase. The identified interaction between emotion and function as well as the presence of ups and downs for each factor would help clinicians to understand the functional progressions and the effect of emotional changes in patients undergoing postoperative rehabilitation.

## 2. Materials and Methods

### 2.1. Participant Selection

Collegiate student-athletes (enrolled in the institutional injury surveillance system) who underwent an orthopedic surgery due to a lower-extremity injury were recruited through convenient sampling between February 2019 and January 2020. A lower-extremity injury was defined as a lower-extremity musculoskeletal condition that required surgery. Surgery was defined as a medical intervention performed by an orthopedic surgeon, aiming to fix or repair injured structures. Once a surgery date was scheduled after consulting with an orthopedic surgeon, patients were informed about the study. After a full understanding of the eligibility criteria and the testing procedures, patients gave written informed consent and determined to participate in the study. The informed consent form and the study procedures were approved by the Institutional Review Board of the Kyung Hee University (KHSIRB-19-001). 

Patients who had surgery in the preceding 48 h prior to obtaining the baseline data collection were excluded. Patients who had the following conditions (either pre-existing or current) for the last six months or who were taking medications due to these conditions were also excluded: (1) any medical conditions (e.g., diabetes, hypertension, neurological disorder) that may influence emotional and functional status and (2) mental disturbance, defined as any health condition affecting emotional and/or behavioral change, other than one from the musculoskeletal condition that met the inclusion criteria.

Eleven patients initially volunteered but seven were subsequently excluded—four were excluded because the baseline data were not obtained within 48 h from surgery, two were discontinued from rehabilitation because of facility shutdown due to the coronavirus disease 2019 pandemic and one transferred to another clinic due to the long distance between home and the rehabilitation clinic. Therefore, four patients were finally analyzed (1 female, 3 males: 21.8 years, 170.4 cm, 68.3 kg: Table 1). 

### 2.2. Testing Procedures 

Initial data were collected in the hospital where the patients underwent surgery and therefore reported to an outpatient clinic where they performed postoperative rehabilitation. After surgery, each patient participated in postoperative rehabilitation under direct supervision of designated clinicians (athletic trainers and physiotherapists), who performed all dependent measurements. Each patient’s number of rehabilitation sessions (number of clinic visits) and time for each rehabilitation session (in minutes) were recorded. Specific therapeutic interventions as classified by six categories (aerobic, flexibility, resistance, plyometrics, balance, and therapeutic modality) were also obtained.

Emotion was recorded using a modified VAS [18] before and after each rehabilitation session. The question ‘how do you feel now?’ was presented above the 100 mm line with the left and right ends labelled ‘worst’ and ‘best,’ respectively. Patients were asked to mark their feelings on the scale. To avoid bias from previous marks, a blank VAS was provided for every measurement and the previously marked VAS was not presented. To quantify emotional status, the distance from the left end to each patient’s mark was measured and rounded to one decimal place. To evaluate self-reported function, the LEFS (score out of 80 points total) [19] was deployed every eight sessions throughout the rehabilitation period. This functional outcome measure contained 20 questions to assess difficulties in daily activities. Patients were asked to mark one answer on a five-point scale (0 through 4). The original scores of the LEFS were converted into 100 points to match the scale of emotional quantification (100 mm).

### 2.3. Statistical Analyses

Different numbers of clinic visits (number of rehabilitation sessions) for each patient yielded different amounts of data points. To conduct statistical analysis, data for emotion and function were session-normalized using a polynomial function [20]. The mean and 95% confidence interval for all data were calculated.

Pearson correlation coefficient (*r*) was calculated to determine the bivariate relationships (*p* < 0.05) among the datasets of the emotion (before and after each session and overall: time-collapsed emotion) and that of function. To examine the presence of ups and downs in each dependent measurement throughout the rehabilitation period, two-way (time × session interaction: emotion) and one-way (session effect: emotion before and after each session and function) analyses of variance (*p* < 0.05) were performed. To determine ups and downs in reference to the values at the first session (and to reduce the type I error), Dunnett’s comparisons were performed as post-hoc tests (*p* < 0.10). To test differences in therapeutic interventions (aerobic, flexibility, resistance, plyometrics, balance, and therapeutic modality) across patients, a chi-square test using binary variables was conducted (*p* < 0.05). To determine practical significance, Cohen’s d effect sizes (ES) were calculated when statistical differences existed [21]. Matlab version 2016a (Mathworks, Natick, MA, USA) and SAS version 9.4 (SAS Institute Inc., Cary, NC, USA) and Microsoft Excel version 2019 (Microsoft Corporation, Redmond, WA, USA) were used.

## 3. Results

**Figure 1:**
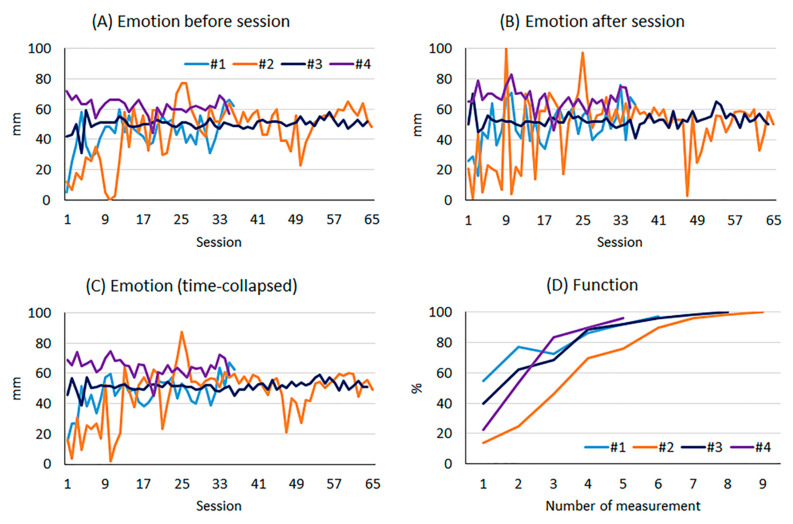
Raw data of emotional changes and functional progressions in patient #1 through #4. Emotional profiles obtained before (**A**) and after (**B**) each session and combined (**C**). The x-axis in (**D**) function indicates the number of functional outcome measurements (Lower-extremity Functional Scale).

**Figure 2:**
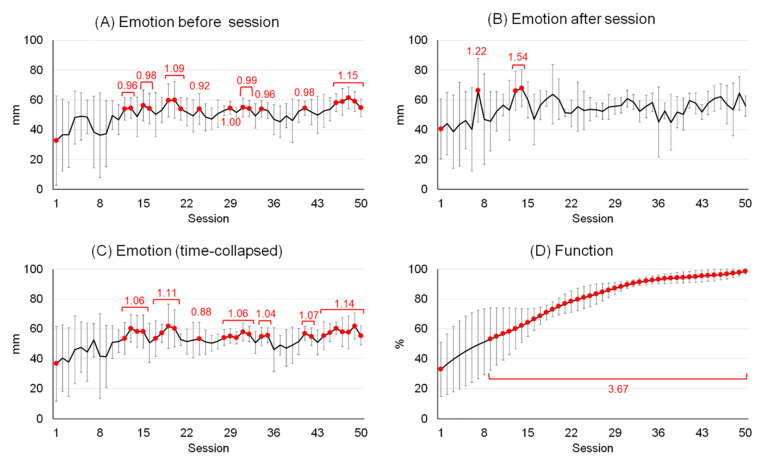
Emotional and functional ups and downs throughout the rehabilitation. All data were session-normalized into 50. Emotional profiles obtained before (**A**) and after (**B**) each session and combined (**C**); functional progressions during rehabilitation (**D**). Values are mean and 95% confidence intervals. The red dots represent statistical differences (post-hoc comparisons using Dunnett’s test) from the first session (*p* < 0.10). The numbers in red font indicate the averaged effect size within the specified period.

Patient demographics, past medical history, and rehabilitation information are presented in Table 1. Recorded raw data for emotion before and after each session and combined (time-collapsed) and those for function are plotted in Figure 1. Based on the average number of rehabilitation sessions (50.0 ± 16.4), each dataset (emotion before and after each session and function) were session-normalized into 50 data points (data points are marked as sessions: Figure 2). 

### 3.1. Correlations between Emotion and Function

All comparisons in the bivariate correlations were statistically significant (*r* values ranged between 0.41 and 0.89: Table 2). There was a moderate correlation between emotion (prior to session: *r* = 0.62, *p* < 0.0001; after session: *r* = 0.41, *p* < 0.01) and function. For overall emotion, the *r* value was 0.58 (*p* < 0.0001).

### 3.2. Ups and Downs of Emotion and Function Throughout the Rehabilitation Period

There was no time effect over session in patient emotion (time × session: F_49,297_ = 0.63, *p* = 0.98). Regardless of session (time effect: F_1,297_ = 10.41, *p* = 0.001), however, overall emotion was improved by 6% after each session of rehabilitation (51 to 54 mm, ES = 0.54). Meanwhile, regardless of time (session effect: F_49,297_ = 2.25, *p* < 0.0001), patient emotions were altered over the rehabilitation period (Figure 2C). After the first improvement at the 12th session (37 to 54 mm, 31%, *p* = 0.07, ES = 0.86), patients experienced six emotional downs throughout rehabilitation (occurring at the 16th, from the 21st to 23rd, from the 25th to 27th, at the 33rd, from the 36th to 40th, at the 43rd session: where the data without red dots in Figure 2C). Considering the emotion data obtained before each rehabilitation session (session effect: F_49,147_ = 1.61, *p* = 0.02: Figure 2A), there were eight emotional downs occurring at the 14th, from the 17th to the 18th, from the 22nd to the 23rd, from the 25th to the 28th, at the 30th, at the 33rd, from the 35th to the 40th and from the 42nd to the 45th session(s) after the first improvement at the 12th session (33 to 54 mm, 39%, *p* = 0.09, ES = 0.95). There was no statistically significant difference among emotions recorded after sessions (session effect: F_49,147_ = 1.30, *p* = 0.12: Figure 2B). 

Self-reported function (session effect: F_49,147_ = 17.39, *p* < 0.0001) consistently progressed after the first improvement (33 points) at the 9th session (53 points, 38%, *p* = 0.07, ES = 1.02) and there was no functional deterioration (Figure 2D).

### 3.3. Rehabilitation

On average, our patients began rehabilitation 2.5 days after their surgery, visited the clinic 50 times (50 sessions of rehabilitation) during the rehabilitation period of 17.2 weeks (2.9 times per week) and participated for approximately two hours (122-min) per rehabilitation session (Table 1). The six components of rehabilitative interventions across the four patients were different (*x*^2^ = 31.3, *df* = 15, *p* < 0.01). Therapeutic modality (29%) and resistance exercise (28%) were the two most frequent treatments, followed by aerobic (16%), balance (11%), flexibility (11%), and plyometrics (5%) exercises. 

## 4. Discussion

Our hypotheses were supported in that emotional changes were moderately correlated with functional progressions; a single-session of rehabilitation resulted in a slight emotional improvement and there was emotional fluctuation along with consistent functional progression. The results of our descriptive data offer a better understanding of how collegiate student-athletes psychologically and physically progress during postoperative rehabilitation. Our study supports that, when patients appear to experience emotional struggle or remain at a low emotional status for a long time period, clinicians could initiate open communication with them in a manner that may have a positive effect. Explaining the interactive relationship between emotion and function and that emotional ups and downs are common during the rehabilitation process could help patients adhere to rehabilitation, achieve emotional improvement and stay at a higher emotional status.

Between emotion and function, there was a moderate (emotion after session vs. function: *r* = 0.41; emotion before session vs. function: *r* = 0.62) correlation (time-collapsed: *r* = 0.58). Our observation is consistent with those of previous reports [22,23] suggesting that more positive emotions (e.g., towards to feeling good or happiness) lead to more positive functional outcomes. To display a matched scale of emotion (a 100 mm VAS), we converted the raw score of function (out of 80 points) into 100 points (Figure 1D and Figure 2D). Although moderately correlated, the variations in emotion and function were much different. When expressed as change in percentage points, the difference between minimum and maximum values in emotion and function were 25% (37–62% for time-collapsed emotion) and 66% (33–98% for time-collapsed emotion), respectively. Given that the minimum values for each factor were recorded at the first session and had similar starting points, our observation suggests that achieving full functional restoration with an only slight emotional improvement may be a common phenomenon among collegiate student-athletes undergoing postoperative rehabilitation. The calculated *r*-values indicate that at least 17% up to 38% of the variation in functional progression can be explained by variation in emotional change. Emotion is difficult to define and/or measure. Overtime, researchers have tested many different approaches with various measurement tools. Emotion can be measured by physiological responses such as autonomic nervous system [24], brain states such as neuroimaging [25] or survey questionnaires such as the Short Form 36 [26], Emotional Reactivity Scale [27], and Depression Anxiety Stress Scale [28]. Those measurements take several minutes to days to obtain the results while our measurement using a VAS took only a few seconds. The reason for choosing the VAS was linked to our research aim to evaluate and quantify emotional changes after each session of rehabilitation. Our measurement was able to capture the present emotional status, which is a valid and recommended method when taking self-reports of emotion [29]. Therefore, using the survey questions listed above was not appropriate for such high measurement frequency. Additionally, VAS is easy and convenient to measure, compare, and interpret patients’ emotional status in the field.

Although statistically significant (a F-value of 10.4 in the time effect with an ES of 0.54), the amount of emotional improvement measured on a VAS was 3 mm (51 to 54 mm). Although limited to quantifying pain, previously established minimal differences on VAS were at least 15 mm [30] or 18 mm [31]. Based on that, the overall degree of emotional improvement after a single session in our study is not clinically meaningful. According to previous studies concerning the effect of a single-session, a 60-min Yoga program in healthy adults [32] or a 5-min hand massage in patients awaiting surgery [33] resulted in emotional improvements of 11% (positive engagement) and 50% (anxiety using the VAS), respectively. In these studies, salutary changes (e.g., breath work) and facilitation of blood circulation were explained as possible mechanisms behind the emotional improvement. We assume that there could have been a similar effect but a relatively smaller amount of emotional improvement in our study compared with those in previous studies [32,33]. This discrepancy could be explained by characteristics of study population (healthy vs. surgery) and the frequency of measurements. While previous reports have examined a single-session effect, a series of repetitive measurements could have resulted in a relatively lower emotional improvement in our study. This idea of measurement adaptation is supported by the differences in emotion before and after rehabilitation: on average, there was an 8 mm improvement for the first 40% of the rehabilitation period (up to the 20th session), with a lower (4 mm) corresponding value during the rest of the period. 

While function consistently improved, emotional fluctuation was observed during the rehabilitation period. The first emotional improvement (time-collapsed) appeared at the time point corresponding to 24% of the rehabilitation period. Afterwards, patients experienced six emotional deteriorations (encompassing a total duration of 37% of the remaining rehabilitation period), which is corresponding to 28% of the entire rehabilitation period (Figure 2C). The emotional downs in the early stage (within the first 25% of the total time) could be due to surgery-related frustration and depression [34]. Meanwhile, the mid-and later-stages, emotional downs could be attributed to lack of motivation due to reduced enthusiasm and adherence over time [2]. In addition to, psychosocial variables such as personality, social and environmental support, and life stress could have also influenced emotional fluctuation [35]. The results of our study were attributed to several unique subject characteristics. Our patients were singles college-age individuals, living in school dormitories with roommates and facing academic issues performing well in classes to maintain scholarships. The emotional responses, therefore, could be combined results of internal (subjective feeling of progression in physical condition) and external (environmental and psychosocial factors associated with college life) factors of rehabilitation.

Several important limitations and assumptions of our study should be addressed. First, our study has a small sample size (*n* = 4), which limits generalization of the results. When considering an expected change in the VAS of 16.8 mm with a standard deviation of 19.1 mm (an ES of 0.88) based on our data, the minimum number of subjects need to detect clinically meaningful effect was calculated as ten (α: 0.05; β: 0.2). Along with the internal and external psychosocial factors above, degree of heterogeneity in terms of gender, family, sport, performance level, previous history of surgery and rehabilitation, characteristics (type, structure and severity) of the lower-extremity pathology or other medical condition in our patients certainly impact our results. Based on the results of this preliminary analysis, data from a larger number of patients from a narrow population should be analyzed in the future. Since emotion is complex, as stated above, it should be measured and analyzed by a multidimensional approach. There are emotionally mediated contributing factors that can potentially affect clinical function. For example, level of certain hormones (e.g., testosterone and cortisol) due to emotional changes can influence the healing process and functional outcomes. Therefore, we acknowledge that our assessment using the VAS cannot fully reflect emotional response. The nature of rehabilitative treatment and communication with clinicians in terms of quantity and quality across patients may have varied, which could have affected the study results. According to the chi-square test, specific therapeutic rehabilitative interventions were different. However, maintaining similar proportions of therapeutic interventions in patients with different pathologies is not possible. The sampling frequency in main outcomes was different in that emotion measurements were obtained each session while function was assessed every eight sessions; as such, the average numbers of measurements for emotion and function were 50 and seven, respectively. For functional outcome measures, however, the time course of change should be considered for determining the optimal measurement frequency [36].

## 5. Conclusions

We observed a moderate linear relationship between emotional changes and functional progression in collegiate student-athletes during the postoperative rehabilitation period. A single session of rehabilitation resulted in slight emotional improvement. After the first emotional improvement, patients experienced six instances of emotional deterioration (37% of the time), while function consistently progressed. The results of our preliminary data have several clinical implications: clinicians should be aware of the relationship between emotion and function and the potential existence of emotional ups and downs throughout the rehabilitation period; psychological consultation focusing on these topics could help patients advance to an emotional improvement and remain at positive emotional status. It should be noted that we only provided preliminary data (*n* = 4), which has a limitation for generalizing the results. Future researchers should conduct a study with a larger number of participants from the homogenous clinical population.

## Figures and Tables

**Table 1:** Patient demographics, past medical history, and rehabilitation information.

Mean ± 95 CIs	Patient #1	Patient #2	Patient #3	Patient #4	Total
Age (year), gender	21, M	23, M	21, F	22, M	21.8 ± 0.8, 3F & 1M
Height (cm), mass (kg)	174, 75	181, 80	157, 51	170, 67	170.4 ± 9.0, 68.3 ± 11.1
Athletic career (year), sport	12, Handball	11, Taekwondo	11, Taekwondo	11, Taekwondo	11.3 ± 0.5, N/A
Diagnosis	R foot fracture	R foot fracture	L ankle sprain	R ankle fracture	-
Surgical procedure	Open reduction internal fixation	Open reduction internal fixation	Ligament repair	Ligament repair; chondroplasty	-
Past surgical history	Yes	Yes	No	No	-
Total # of clinic visits	36	65	64	35	50.0 ± 16.4
# of visits per weeks	2.5	3.5	3.2	2.2	2.9 ± 0.6
Rehabilitation time (min)	144.7 ± 12.1	120.3 ± 8.8	128.1 ± 8.8	96.6 ± 15.0	122.4 ± 19.6
Specific interventions (%)					
Aerobic	6.7	21.5	17.8	18.2	16.1 ± 6.3
Flexibility	18.1	5.7	10.2	9.1	10.8 ± 5.1
Resistance	32.4	29.7	25.4	25.3	28.2 ± 3.4
Plyometrics	0	9.1	8.5	3.0	5.2 ± 4.3
Balance	9.5	9.1	12.7	14.1	11.4 ± 2.4
Therapeutic modality	33.3	24.9	25.4	30.3	28.5 ± 4.0

CI: confidence intervals; F: female; L: left: M: male; N/A: not applicable; R: right. Past surgical history: patient #1 had the right fifth metatarsal fracture; patient #2 had the right fourth finger laceration and right Achilles tendon rupture. # of visits per weeks= total # of clinic visits/total weeks of rehabilitation period; Specific interventions = total # of the specific interventions administered/total # of all interventions administered × 100.

**Table 2:** Bivariate correlation among dependent measurements.

	Emotion Before Session	Emotion After Session	Emotion (Time-Collapsed)	Function
Emotion Before Session	1			
Emotion After Session	0.56 *	1		
Emotion (time-collapsed)	0.88 *	0.89 *	1	
Function	0.62 *	0.41 ‡	0.58 *	1

Pearson correlation coefficient = *r* value, * *p* < 0.0001, ‡ *p* < 0.01

## Data Availability

The data are not publicly available due to privacy or ethical restrictions.
